# Factors associated with the length of breastfeeding during the COVID-19 pandemic: a survival study

**DOI:** 10.1590/1980-220X-REEUSP-2024-0078en

**Published:** 2024-08-16

**Authors:** Thales Philipe Rodrigues da Silva, Lorrayne Oliveira Dias Soares, Luana Carolina dos Santos, Fernanda Marçal Ferreira, Rafaela Siqueira Costa Schreck, Renata Melgaço Gonçalves, Gabriela Muniz Vidigal dos Santos, Fernanda Penido Matozinhos

**Affiliations:** 1 Universidade Federal de São Paulo, Escola Paulista de Enfermagem, Departamento de Enfermagem na Saúde da Mulher, São Paulo, SP, Brazil.; 2 Universidade Federal de Minas Gerais, Escola de Enfermagem, Departamento de Enfermagem Materno Infantil e Saúde Pública, Belo Horizonte, MG, Brazil.; 3 Universidade Federal de Minas Gerais, Escola de Enfermagem, Departamento de Nutrição, Belo Horizonte, MG, Brazil.; 4 Universidade de São Paulo, Escola de Enfermagem, Departamento de Enfermagem Materno-Infantil e Psiquiátrica, São Paulo, SP, Brazil.; 5 Universidade Federal de Minas Gerais, Escola de Enfermagem, Programa de Pós-Graduação em Enfermagem, Belo Horizonte, MG, Brazil.; 6 Faculdade de Ciências Médicas de Minas Gerais, Belo Horizonte, MG, Brazil.

**Keywords:** Pandemics, Breast Feeding, Weaning, Nursing, Pandemias, Lactancia Materna, Destete, Enfermería, Pandemias, Aleitamento Materno, Desmame, Enfermagem

## Abstract

**Objective:**

To investigate the repercussions of COVID-19 on the length of breastfeeding and analyze the associated factors in Belo Horizonte, Minas Gerais, Brazil.

**Method:**

This is an epidemiological, prospective cohort study. Data were collected from medical records and through telephone interviews. Women who weaned were estimated using Kaplan-Meier survival analysis. The log-rank test was used to verify differences between groups, analyzing weaning time, according to sociodemographic and clinical characteristics. The values of hazard ratio and 95% confidence intervals were estimated using Cox regression analysis.

**Results:**

A total of 1,729 women participated in the study. During the COVID-19 pandemic, brown women and women undergoing cesarean section were more likely to stop breastfeeding.

**Conclusion:**

The birth route and mothers’ ethnic characteristics were associated with early weaning during the COVID-19 pandemic. Such findings are important to guide the assistance of the multidisciplinary team, especially nursing, during the post-pandemic period and in future epidemiological scenarios.

## RESUMO

**Objetivo:**

Investigar as repercussões da COVID-19 na duração do aleitamento materno e analisar os fatores associados em Belo Horizonte, Minas Gerais, Brasil.

**Método:**

Trata-se de estudo epidemiológico, de coorte prospectiva. Os dados foram coletados em prontuários médicos e por meio de entrevistas telefônicas. As mulheres que apresentaram o desmame foram estimadas por meio da análise de sobrevivência de Kaplan-Meier. O teste *log-rank* foi utilizado para verificar diferenças entre os grupos, analisando-se o tempo de desmame, segundo as características sociodemográficas e clínicas. Os valores de *hazard ratio* e intervalos de confiança de 95% foram estimados utilizando-se análise de regressão de Cox.

**Resultados:**

Participaram da pesquisa 1.729 mulheres. Durante a pandemia de COVID-19, mulheres pardas e mulheres submetidas à cesariana foram mais suscetíveis a interromper a amamentação.

**Conclusão:**

A via de nascimento e as características étnicas das mães associaram-se ao desmame precoce durante a pandemia de COVID-19. Tais achados são importantes para nortear a assistência da equipe multiprofissional, sobretudo da enfermagem, durante o pós-pandemia e em cenários epidemiológicos futuros.

## RESUMEN

**Objetivo:**

Investigar las repercusiones de la COVID-19 en la duración de la lactancia materna y analizar los factores asociados en Belo Horizonte, Minas Gerais, Brasil.

**Método:**

Se trata de un estudio de cohorte prospectivo epidemiológico. Los datos se recogieron de historias clínicas y mediante entrevistas telefónicas. Las mujeres que destetaron se estimaron mediante el análisis de supervivencia de Kaplan-Meier. La prueba de Mantel-Cox se utilizó para verificar diferencias entre grupos, analizando el tiempo de destete, según características sociodemográficas y clínicas. Los valores de cociente de riesgo y los intervalos de confianza del 95% se estimaron mediante análisis de regresión de Cox.

**Resultados:**

Un total de 1.729 mujeres participaron en la investigación. Durante la pandemia de COVID-19, las mujeres pardas y aquellas sometidas a cesárea tenían más probabilidades de dejar de amamantar.

**Conclusión:**

La ruta de nacimiento y las características étnicas de las madres se asociaron con el destete temprano durante la pandemia de COVID-19. Tales hallazgos son importantes para orientar la asistencia del equipo multidisciplinario, especialmente de enfermería, durante el período pospandemia y en futuros escenarios epidemiológicos.

## INTRODUCTION

Breastfeeding, in addition to reducing infant mortality and strengthening the immune system, provides numerous other benefits to the immediate infant’s health, such as: adequate development of the stomatognathic system and prevention of respiratory infections^(1,2)^. The advantages of the practice are also associated with a reduction in the occurrence, in adulthood, of diabetes mellitus, high blood pressure, and obesity^(3,4)^. Breastfeeding can also indirectly impact adults’ income and intellectual levels^(4)^.

Furthermore, breastfeeding is a practice that also benefits the nursing mother, as it is associated with a reduction in the incidence of breast, ovarian, and endometrial cancers, osteoporosis, multiple sclerosis, diabetes mellitus, high blood pressure, and cardiovascular diseases, and with strengthening of the mother-child emotional bond^(5,6)^.

For these reasons, the World Health Organization (WHO) recommends that breast milk be the exclusive food (EBF) until 6 months of age and up to 24 months of age, at least, as a complementary food^(2)^. Despite the advantages, the prevalence of breastfeeding in Brazil is far below the targets established by the WHO for the year 2030: the WHO recommends a prevalence of at least 60% of EBF, and the National Study of Child Food and Nutrition, carried out in 2019 by the Universidade Federal do Rio de Janeiro (UFRJ) indicates that only 35.5% of Brazilian children aged between 20 and 23 months continue breastfeeding^(7)^.

This is explained by the fact that breastfeeding is a complex process and involves not only individual factors, but also family, cultural, historical-geographical and socioeconomic reasons, among others^(8)^. In addition, COVID-19, a respiratory disease caused by the SARS-Cov-2 virus, has become an important influencing factor for breastfeeding^(9)^. Some studies show benefits of the pandemic for breastfeeding^(10,11)^, as demonstrated by a study carried out in the United States, which highlighted the positive effects of the measures of social isolation and staying at home on breastfeeding practices, specifically in this country where there are no remuneration policies during the period of maternity leave^(12)^. However, in general, the pandemic was an influential circumstance for early weaning^(13)^, especially when related to maternal mental health^(14)^ and social and economic inequalities^(15)^. In this regard, a systematic review identified the negative effects of the COVID-19 pandemic, mainly for family support, with influences on the continuity of breastfeeding^(16)^.

In Brazil, there are few studies related to the topic addressed in this work. Considering the importance of breastfeeding for promoting maternal and child health and the repercussions of COVID-19 to the present day, which may have influenced the duration and maintenance of breastfeeding (exclusive or mixed), the identification of the incidence of weaning after SARS-COV-2 infection and its associated factors is required, aiming at contributing to future strategies that allow for better maintenance of breastfeeding.

The hypothesis of this study is that the pandemic influenced the duration and maintenance of breastfeeding. The objective of this study was to investigate the repercussions of COVID-19 on the length of breastfeeding and analyze the associated factors in Belo Horizonte, Minas Gerais, Brazil.

## METHOD

### Design of Study

This is an epidemiological, prospective cohort study, which had as its setting three hospitals that provide their services exclusively through the Brazilian Public Health System – *SUS*, in Belo Horizonte (BH), Minas Gerais (MG), Brazil, which are participants in the Research entitled “Childbirth and breastfeeding in children of mothers infected with SARS-CoV-2”.

### Population, Local and Selection Criteria

Postpartum women with a single pregnancy were included, whose delivery was in a hospital and had as product their live newborn conceptuses (NB) aged 22 weeks or more, weighing more than 500 grams at birth. They were women admitted to the three maternity hospitals selected at the time of delivery and who went into labor (L) (induced or not) and whose birth was vaginal or C-section.

### Sample Definition

For the sample calculation, the cohort study design was considered. A ratio of nine pregnant women in the unexposed group (without COVID-19) for each pregnant woman in the exposed group (with COVID-19) was considered, given the infection rate of 10% during the epidemic period^(17)^. This was the proportion considered for the event in the unexposed group. Furthermore, *Odds Ratio* of 1.5 was estimated, for a confidence level of 95% and power of 80%. The distribution of the number of pregnant women by participating maternity hospitals followed the proportion of the total number of births in each selected maternity hospital. The sample was selected randomly, through a simple draw, until reaching the number of postpartum women for each hospital, totaling 1,729 postpartum women. As this was a prospective cohort study, the women were contacted 6 months after delivery to investigate EBF via telephone. In this process, 410 postpartum women were accessed. It should be noted those women who could not be reached through telephone were considered lost to follow-up.

### Data Collection

The study data were collected from the medical records of hospital institutions, using a semi-structured questionnaire adapted from the research “Born in Belo Horizonte: Inquiry into labor and birth (Nascer em Belo Horizonte: Inquérito sobre o parto e nascimento”. The medical records of all women who had their children in the respective hospitals in the three months with the highest incidence of COVID-19 (May, June and July) in the first half of 2020, in Brazil, were analyzed.

After collecting the medical records, the postpartum women were contacted via telephone call after 2 months of delivery. They were also contacted 6 months after birth to investigate EBF. As a research protocol, the postpartum women were accessed at different times and telephone contact was made at least 5 times by trained researchers. In case of refusal or failure in these attempts, the postpartum woman was excluded.

The following variables were considered: sociodemographic (age, education, income, marital status, race/skin color), clinical (current/previous illnesses and previous surgeries, use of medication, symptoms and signs of SARS-COV-2), health status (smoking, alcohol consumption, intestinal habits, physical activity, monitoring with a health professional and vaccination), obstetric (number of births, route of birth, presence of a companion, practices and interventions during birth, reproductive outcome, and complications of the last delivery, support network, changes in care due to the COVID-19 pandemic, information about the occurrence of SARS-CoV-2 infection/symptoms in the participant and her child, including separation of the binomial), and specific information about breastfeeding (type, difficulties, interference of the COVID-19 pandemic in breastfeeding, milk donation, breastfeeding in the first hour of life). In relation to difficulty in breastfeeding, this variable was verified through reports from the postpartum woman about the occurrence of some “nipple trauma, hard, red, bruised breast, or need to use antibiotics”. Regarding the interference of the COVID-19 pandemic, this variable was according to the perception of the postpartum woman.

### Data Analysis and Treatment

To analyze the association between sociodemographic and clinical characteristics with EBF, the statistical package *Statistics Software for Data Science* (Stata), version 16.0 was used. Initially, the population was described, and estimates were presented in proportions (%), with 95% CI. For quantitative variables, after asymmetry was verified using the Shapiro-Wilk test, data were presented using median and interquartile range (IQ).

The non-parametric statistical method of survival curves was used to estimate the fraction of women who weaned, using Kaplan-Meier estimates.

The log-rank test was used to check for the presence of differences between groups (weaning yes and no; p < 0.05), analyzing weaning time, according to sociodemographic and clinical characteristics.

Additionally, Hazard Ratio (HR) values and unadjusted 95% confidence intervals (95% CI) were estimated, using the Cox proportional hazards model. At the end, the adjusted analysis of the multivariate model was carried out, with the inclusion of variables that had a significance of up to 0.2 in the unadjusted analysis. The forward method was used, with one-by-one variables input following the decreasing level of significance.

### Ethical Aspects

The study “Birth and breastfeeding in children of mothers infected by SARS-CoV-2 (Parto e aleitamento materno em filhos de mães infectadas por SARS-CoV-2)” was approved in 2022 by the Research Ethics Committee of the Universidade Federal de Minas Gerais (Opinion Number: 5.735.679).

## RESULTS

In this study, 1,729 women were included. Table 1 shows the sociodemographic and clinical characteristics of the sample. Women’s median age was 28 years (IQ: 23-33), 88.65% had NBs with weight above 2,499 grams, 98.27% had no SAR-CoV-2 virus infection/suspicion. The majority of women were multiparous (62.32%) and had no clinical/obstetric complications (53.77%). Regarding the method of birth, the majority had their children vaginally (72.67%). Finally, there were higher proportions of women who had more than 6 prenatal consultations (77.50%).

**Table 1:** Demographic, socioeconomic, and obstetric profile of the sample of postpartum women – Belo Horizonte, MG, Brazil, 2020/2023 (n = 1729).

Variables	n (%)	95%CI
**Age**	28(23–33)	
**NB weight**		
Above 2499	1359(88.65)	86.96–90.14
Less than 2500	174(11.35)	09.85–13.03
**SARS-CoV-2 infection**		
No	1651(98.27)	97.52–98.79
Yes	29(01.72)	01.20–02.47
**Parity**		
Primiparous	633(37.68)	35.38–40.02
Multiparous	1047(62.32)	59.97–64.61
**Clinical/obstetric complications**		
No	813(53.77)	51.24–56.27
Yes	699(46.23)	43.72–48.75
**Presence of companion during birth**		
Yes	1225(89.68)	87.94–91.18
No	141(9.41)	08.81–12.05
**Birth route**		
Vaginal	1210(72.67)	70.47–74.76
C-section	455(27.33)	25.32–29.52
**NB complication**		
No	1188(77.90)	75.74–79.91
Yes	337(22.10)	20.08–24.25
**Number of PN consultations**		
More than or equal to 6 visits	589(77.50)	74.38–80.33
Less than 6 visits	171(22.50)	19.66–25.61

**Notes:** ^1^Median (IQ); NB: newborn; PN: prenatal.

**Figure 1:**
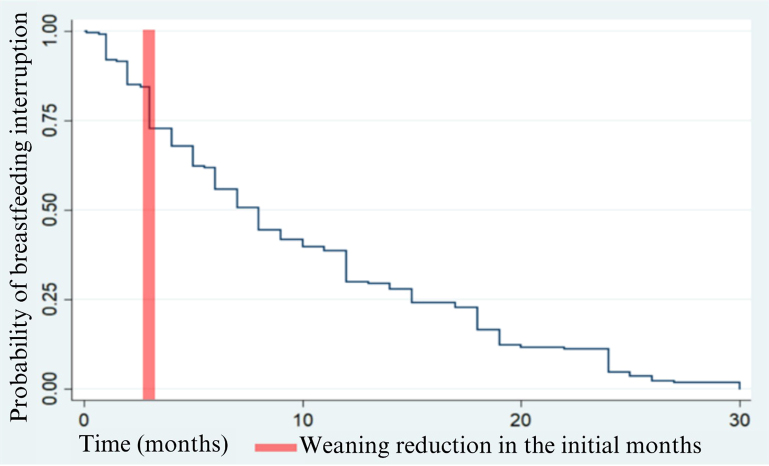
Kaplan-Meier curve relating the probability of interrupting breastfeeding (vertical axis) as a function of time in months (horizontal time).

In Figure 1, the Kaplan-Meyer curve of probability of weaning survival is represented. During follow-up, 410 women were accessed and, of these, 53.66% weaned. The survival function (t) is represented on the vertical axis and the survival time (T), in months, on the horizontal axis. It indicates the probability that a woman will wean during a specific period of time. It is possible to state that the longer the time, the lower the probability of weaning, that is, weaning occurred mainly in the initial months after birth. Regarding the incidence of the weaning event, it was 99.41 per 1000 person-days (95%CI 86.36 – 114.43).

**Figure 2:**
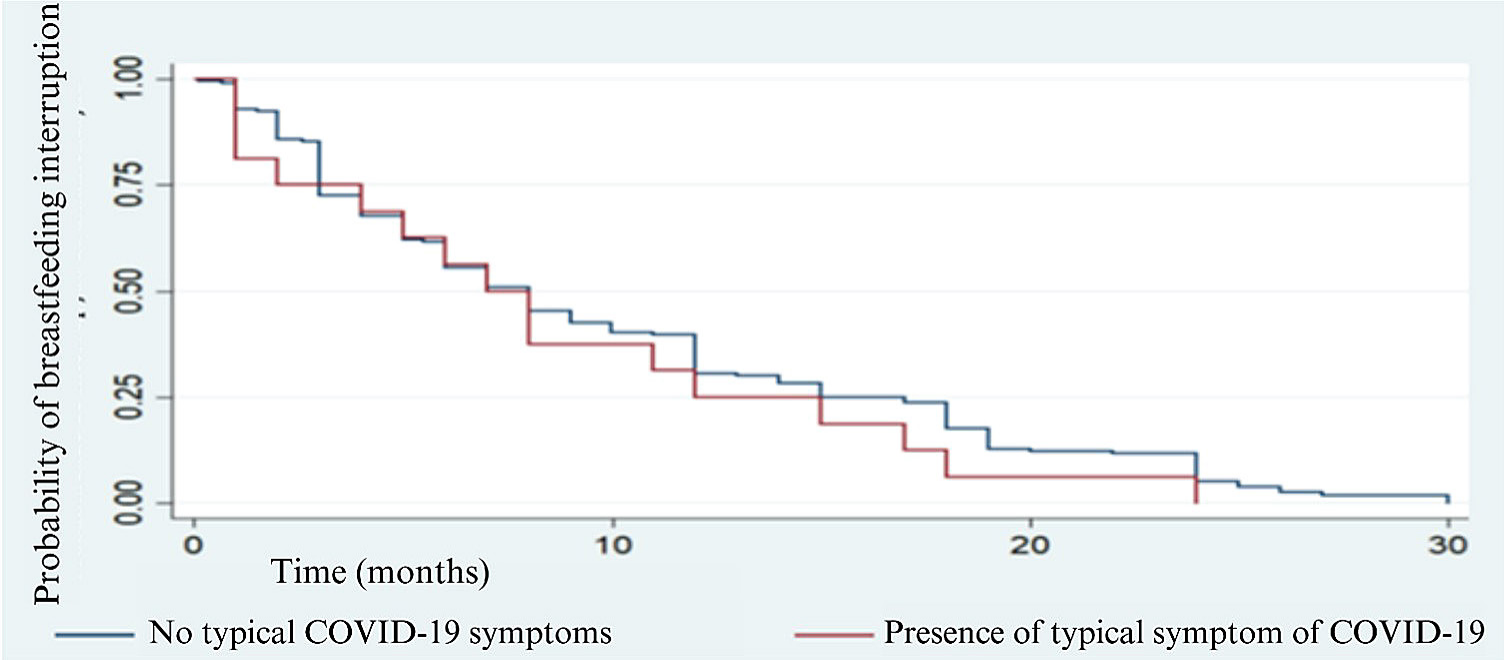
Probability of breastfeeding interruption without typical COVID-19 symptoms(blue) and with typical symptom of COVID-19 during breastfeeding (red).

Figure 2, in its turn, demonstrates the probability of a weaning event in women who did or did not show symptoms or signs of COVID-19, with no statistical significance for the log-rank test (p = 0.348) when comparing the groups.

Table 2 shows the weaning incidence rates (events/1000 person-days), according to the sample sociodemographic and clinical variables. A statistically significant difference was observed through the log-rank test for the interruption of breastfeeding when considering the self-reported skin color and the newborn’s birth route.

**Table 2:** Weaning incidence rates (events/1000 person-days), according to sociodemographic and clinical variables – Belo Horizonte, MG, Brazil, 2020/2023 (n = 410).

	Weaning		
	Incidence	95%CI	p-value
**Level of education**			0.337
Higher	81.60	(55.56–119.85)	
High School	99.52	(82.49–120.08)	
Elementary	109.74	(85.03–141.64)	
Income*			0.684
No income	101.76	(82.67–125.25)	
Less than 1 minimum wage	93.54	(74.48–117.47)	
Between 1 and 3 minimum wages	108.51	(76.31–154.30)	
**Lives with partner**			0.759
Yes	97.46	(77.72–122.22)	
No	100.68	(84.13–120.50)	
**Postpartum consultation**			0.981
Yes	99.22	(84.59–116.38)	
No	96.94	(71.11–132.16)	
**Nipple trauma**			0.355
Yes	92.89	(74.61–115.65)	
No	102.31	(84.18–124.34)	
**Breast engorgement**			0.759
Yes	111.76	(71.29–175.22)	
No	108.68	(83.02–142.25)	
**Interferences due to COVID-19**		0.105
Yes	76.27	(52.30–111.21)	
No	104.19	(89.36–121.48)	
**Self-declared skin color**		**0.0355**
White	87.67	(63.52–121.01)	
Black	80.11	(58.76–109.22)	
Brown	113.58	(94.75–136.14)	
**Birth route**			**0.0459**
Vaginal	93.72	(79.69–110.23)	
Cesarean section	121.93	(91.88–161.79)	

**Notes:** *The minimum wage in force at the time was R$1,039.00; p-value in bold: statistical significance for the log-rank test.

## DISCUSSION

The general results of this study demonstrated that characteristics such as self-reported skin color and the newborn’s birth route were influencing factors in weaning rates during the COVID-19 pandemic.

Regarding the method of birth, women undergoing cesarean section were more likely to interrupt breastfeeding. In this regard, the strong influence of cesarean sections on difficulties in the breastfeeding process or early weaning is recognized, since in this type of surgery newborns are less exposed to skin-to-skin contact and the effect of anesthesia restricts the practice of breastfeeding in the first hour of life^(18,19)^. Furthermore, cross-sectional epidemiological studies carried out in Brazil found a significant increase in the percentage rates of cesarean sections, with a possible influence of the pandemic on the indications for cesarean sections at the time of admission to the maternity ward^(20,21)^.

For self-reported skin color, brown women had higher rates of early weaning. The association between prenatal care process indicators and the race/color of women demonstrates that black and mixed-race women are more exposed to access difficulties and inadequate prenatal care^(22,23)^. Regarding guidance on breastfeeding during pregnancy, childbirth, and postpartum, a population-based cross-sectional study shows that black and mixed-race pregnant women had a 33% lower chance of receiving guidance on the subject^(24)^.

The increase in social inequalities in health during the pandemic period is highlighted, especially regarding access to services^(25,26)^. In Brazil, the impact of the pandemic has highlighted existing racial disparities in health. In this context, an integrative review using population databases observed that, in Brazil, being black or brown was a risk factor for the worsening of COVID-19^(27).^

Notwithstanding, there is a lack of research on mothers’ self-declared skin color as a factor associated with early weaning during the pandemic. Research carried out in the United Kingdom found that black and brown women, when compared to white women, felt a greater negative impact of social isolation measures on breastfeeding. The study also recognized that black and brown women were less likely to feel they had enough support to continue breastfeeding^(14)^.

Despite some research carried out in Israel^(28)^, Chile^(29)^, Europe and South America^(30)^ concluded that breastfeeding rates were not negatively impacted by the COVID-19 pandemic, most studies on the subject concluded that the pandemic had a negative influence on breastfeeding rates^(9,14)^ and those that identified maternal psychological factors as determinants for weaning^(12,13)^.

Regarding the latter approach, research carried out in Thailand concluded that psychological factors were the preponderant influences for weaning during the pandemic^(31)^. Therefore, depression^(13,32)^, fear^(33,34)^, and anxiety^(35)^ were maternal feelings related to early weaning. This approach, however, differs from the scope of the present study and hinders a real discussion of the results.

Regarding socioeconomic aspects, education levels were the factors most related to weaning during the pandemic. Research carried out in the United Kingdom concluded that women with lower levels of education were more likely to stop breastfeeding^(14)^. Likewise, research carried out in 17 European countries concluded that mothers with higher levels of education were less likely to stop breastfeeding^(12)^. These conclusions are also shared by surveys carried out online^(36)^ and in 5 countries, including Brazil^(37)^.

The present study was unable to relate educational levels to breastfeeding interruption. The discrepancy in relation to the results of the aforementioned research is possibly explained by the different socioeconomic reality of the populations studied.

In the literature, the association of income with the length of breastfeeding during the pandemic period is also recurrent. A study carried out in Thailand reported that women who considered their family income sufficient were also more likely to breastfeed exclusively at six months^(38)^. Another study also concluded that low-income women were more likely to stop breastfeeding due to the greater likelihood of being food insecure^(36)^.

However, in the present study, no association was found between income levels and breastfeeding duration. The difference in results may be related, again, to the different socioeconomic reality of the populations studied. Brazil, during the pandemic, instituted a minimum income program for the most vulnerable, the Emergency Aid, as a way to mitigate the economic impacts of the pandemic. These resources probably had a positive impact, mitigating the effects of food insecurity in the population studied^(38,39)^.

Among the limitations of the research, it is recognized that it was carried out using data obtained from maternity hospitals located in Belo Horizonte. Its conclusions, therefore, only reflect a specific reality and should not be extended to other locations, especially when considering the extensive Brazilian sociocultural diversity and the fact that these maternity hospitals are references in relation to the obstetric model of labor and birth. Another limitation is the possible memory bias of the outcome variable in these studies; thus, the findings found here should be interpreted with caution. However, it should be noted that data are consistent with the Brazilian literature, which demonstrates a prevalence of only 61.3% of EBF at 4 months^(7)^. It is worth highlighting that, in the Brazilian scenario, due to the reduced number of tests for COVID-19, only parturient women who were admitted to hospitals with symptoms and signs of COVID-19 underwent confirmatory tests. Therefore, the Brazilian scenario did not adopt universal testing for all parturient women.

The research scenario was only maternity hospitals that serve exclusively *SUS* and data collection took place through telephone calls. Such facts bring, in themselves, a previous social outline, either because they exclude the higher classes that typically use private health services, or because they exclude from the sample lower class women who may not have access to telephone devices.

## CONCLUSION

In this study, the birth route and mothers’ ethnic characteristics were associated with early weaning during the COVID-19 pandemic. Such findings are important to guide the assistance of the multidisciplinary team, especially the nursing team, during the post-pandemic period and in future or recurring critical epidemiological scenarios.

## References

[1] Braga MS, da Silva Gonçalves M, Augusto CR (2020). Os benefícios do aleitamento materno para o desenvolvimento infantil. Braz. J. Develop.

[2] Ministério da Saúde (BR) (2015). Secretaria de Atenção à Saúde. Departamento de Atenção Básica. Saúde da criança: aleitamento materno e alimentação complementar [Internet]..

[3] Fialho FA, Dias IMAV, Leal DT, do Nascimento L, Neves PM (2014). Almeida MJGG. Diabetes mellitus: a possível relação com o desmame precoce. Rev Enferm UFPE On Line.

[4] Victora CG, Horta BL, de Mola CL, Quevedo L, Pinheiro RT, Gigante DP (2015). Association between breastfeeding and intelligence, educational attainment, and income at 30 years of age: a prospective birth cohort study from Brazil. Lancet Glob Health.

[5] Ribeiro JM, Pereira SE (2021). Benefícios a longo prazo na saúde da mulher promovidos pelo aleitamento materno: uma revisão narrativa [monography]. Goiás: Escola de Ciências Sociais e da Saúde, Pontifícia Universidade Católica de Goiás.

[6] Viana RMS, Cassino L (2017). Aleitamento materno: fortalecedor do vínculo afetivo entre mãe e filho. Rev. Bras. Ci. Vida.

[7] Universidade Federal do Rio De Janeiro (UFRJ) (2021). Aleitamento materno: prevalência e práticas entre crianças brasileiras menores de 2 anos [Internet].

[8] Silva FMP, Nunes HHM, de Almeida JM, de Menezes LDM, Figueiredo ACB, Cardoso ATS (2022). Aspectos culturais relacionados ao aleitamento materno exclusivo em puérperas atendidas em alojamento conjunto. REAS.

[9] Holand BL, de Oliveira Agostini C, Pacheco MCM, de Leon DMZ, Drehmer M, Bosa VL (2022). Association between breastfeeding and complementary feeding in pre-pandemic and pandemic COVID-19 times: maternar cohort study. J Pediatr (Rio J).

[10] Badr H, Alghamdi S (2022). Breastfeeding experience among mothers during the COVID-19 pandemic. Int J Environ Res Public Health.

[11] Kwan J, Jia J, Yip KM, So HK, Leung SSF, Ip P (2022). A mixed-methods study on the association of six-month predominant breastfeeding with socioecological factors and COVID-19 among experienced breastfeeding women in Hong Kong. Int Breastfeed J.

[12] Palmquist AEL, Tomori C, Tumlinson K, Fox C, Chung S, Quinn EA (2022). Pandemic policies and breastfeeding: a cross-sectional study during the onset of COVID-19 in the United States. Front Sociol.

[13] Chertok IA, Artzi-Medvedik R, Arendt M, Sacks E, Otelea MR, Rodrigues C (2022). Factors associated with exclusive breastfeeding at discharge during the COVID-19 pandemic in 17 WHO European Region countries. Int Breastfeed J.

[14] Chang YS, Li KMC, Chien LY, Lee EY, Hong SA, Coca KP (2023). Associations between breastfeeding intention, breastfeeding practices and post-natal depression during the COVID-19 pandemic: a multi-country cross-sectional study. Matern Child Nutr.

[15] Brown A, Shenker N (2021). Experiences of breastfeeding during COVID-19: lessons for future practical and emotional support. Matern Child Nutr.

[16] Lubbe W, Niela-Vilén H, Thomson G, Botha E (2022). Impact of the COVID-19 pandemic on breastfeeding support services and women’s experiences of breastfeeding: a review. Int J Womens Health.

[17] Perlman J, Oxford C, Chang C, Salvatore C, Di Pace J (2020). Delivery room preparedness and early neonatal outcomes during COVID-19 pandemic in New York City. Pediatrics.

[18] Medeiros ACLL, da Silva GSV, Gomes NF, Silva JSLG, Souza AS, da Silva EA (2021). A influência do tipo de parto no desmame precoce. Revista Pró-UniverSUS.

[19] Ferrari AP, Almeida MAM, Carvalhaes MABL, Parada CMGL (2020). Effects of elective cesarean sections on perinatal outcomes and care practices. Rev Bras Saúde Mater Infant.

[20] Oliveira ICDP, Geraldo LMCS, Faria APV, Silva TPRD, Amorim T, Pereira PF (2023). Repercussões da infecção por SARS-CoV-2 e da pandemia nas vias de nascimento: estudo transversal. Rev Gaúcha Enferm.

[21] da Silva CEB, Guida JPS, Costa ML (2023). Increased cesarean section rates during the COVID-19 pandemic: looking for reasons through the robson ten group classification system. Rev Bras Ginecol Obstet.

[22] Lessa MSDA, Nascimento ER, Coelho EDAC, Soares IDJ, Rodrigues QP, Santos CADST (2022). Pré-natal da mulher brasileira: desigualdades raciais e suas implicações para o cuidado. Cien Saude Colet.

[23] Flores TR, Neves RG, Mielke GI, Bertoldi AD, Nunes BP (2021). Desigualdades na cobertura da assistência pré-natal no Brasil: um estudo de abrangência nacional. Cien Saude Colet.

[24] Lessa MSDA, Nascimento ER, Coelho EDAC, Soares IDJ, Rodrigues QP, Santos CADST (2022). Pré-natal da mulher brasileira: desigualdades raciais e suas implicações para o cuidado. Cien Saude Colet.

[25] Dantas-Silva A, Santiago SM, Surita FG (2023). Racism as a social determinant of health in Brazil in the COVID-19 pandemic and beyond. Rev Bras Ginecol Obstet.

[26] Barron GC, Laryea-Adjei G, Vike-Freiberga V, Abubakar I, Dakkak H, Devakumar D (2022). Lancet Commission on COVID-19: task force on humanitarian relief, social protection and vulnerable groups. Safeguarding people living in vulnerable conditions in the COVID-19 era through universal health coverage and social protection. Lancet Public Health.

[27] Cavalcante GS, Santos MC, Andrade MM, Melo RB, Oliveira TS, Santos GG (2021). Revisão Integrativa da Literatura sobre disparidades étnico-raciais da COVID-19 entre gestantes e puérperas negras. Pubsaúde.

[28] Magnazi MB, Sartena G, Goldberg M, Zimmerman D, Ophir E, Baruch R (2022). Impact of the COVID-19 pandemic on breastfeeding in Israel: a cross- sectional, observational survey. Int Breastfeed J.

[29] Navarro-Rosenblatt D, Benmarhnia T, Bedregal P, Lopez-Arana S, Rodriguez-Osiac L, Garmendia ML (2023). Socio-economic inequalities in the effect of public policies and the COVID-19 pandemic on exclusive breastfeeding in Chile. Public Health.

[30] Ganho-Ávila A, Guiomar R, Sobral M, Pacheco F, Caparros-Gonzalez RA, Diaz-Louzao C (2023). The impact of COVID-19 on breastfeeding rates: an international cross-sectional study. Midwifery.

[31] Nuampa S, Patil CL, Prasong S, Kuesakul K, Sudphet M (2022). Exploring the association between socioeconomic and psychological factors and breastfeeding in the first year of life during the COVID-19 pandemic in Thailand. Int J Environ Res Public Health.

[32] Ahmad Zadeh Beheshti M, Alimoradi Z, Bahrami N, Allen KA, Lissack K (2022). Predictors of breastfeeding self-efficacy during the Covid-19 pandemic. J Neonatal Nurs.

[33] Reagu SM, Abuyaqoub S, Babarinsa I, Kader NA, Farrell T, Lindow S (2022). Impact of the fear of Covid-19 infection on intent to breastfeed; a cross sectional survey of a perinatal population in Qatar. BMC Pregnancy Childbirth.

[34] Samaria D, Marcelina LA, Florensia L (2023). The COVID-19 pandemic’s impact on breastfeeding self-efficacy: a path analysis. Enferm Clin.

[35] Ergün S, Kaynak S, Aydın B (2022). Fear of COVID-19 and related factors affecting mothers’ breastfeeding self-efficacy during the pandemic. Rev Esc Enferm USP.

[36] Chien LY, Lee EY, Coca KP, Paek SC, Hong SA, Chang YS (2022). Impact of COVID-19 on breastfeeding intention and behaviour among postpartum women in five countries. Women Birth.

[37] Nuampa S, Ratinthorn A, Patil CL, Kuesakul K, Prasong S, Sudphet M (2022). Impact of personal and environmental factors affecting exclusive breastfeeding practices in the first six months during the COVID-19 pandemic in Thailand: a mixed-methods approach. Int Breastfeed J.

[38] Freire D, Domingue E, Magalhães A, Simonato T, Cardoso G (2021). “Auxílio Emergencial – Uma política fiscal contracíclica?”: Impactos do auxílio emergencial na economia brasileira em 2020 [Internet].

[39] Ramos CL (2021). O impacto do auxílio emergencial sobre a pobreza e a desigualdade durante a pandemia do coronavírus [dissertation].

